# Comparison of endoscopic ultrasound-guided choledochoduodenostomy and endoscopic retrograde cholangiopancreatography in first-line biliary drainage for malignant distal bile duct obstruction: A multicenter randomized controlled trial

**DOI:** 10.1097/MD.0000000000025268

**Published:** 2021-03-26

**Authors:** Masahiro Itonaga, Masayuki Kitano, Takanori Yoshikawa, Reiko Ashida, Yasunobu Yamashita, Kenichi Hatamaru, Mamoru Takenaka, Tomohiro Yamazaki, Takeshi Ogura, Nobu Nishioka, Arata Sakai, Atsuhiro Masuda, Hideyuki Shiomi, Toshio Shimokawa

**Affiliations:** a Second Department of Internal Medicine; b Clinical Study Support Center, Wakayama Medical University, Wakayama; c Department of Gastroenterology and Hepatology, Kindai University Faculty of Medicine, Osaka; d The Second Department of Internal Medicine, Osaka Medical College, Takatsuki; e Division of Gastroenterology, Department of Internal Medicine, Kobe University Graduate School of Medicine, Kobe, Japan.

**Keywords:** ERCP, EUS-BD, EUS-CDS, malignant distal bile duct obstruction

## Abstract

**Introduction:**

In patients with malignant distal bile duct obstruction and normal gastrointestinal anatomy, endoscopic ultrasound-guided choledochoduodenostomy (EUS-CDS) is indicated when endoscopic retrograde cholangiopancreatography (ERCP) fails. The ERCP drainage route passes through the tumor, whereas the EUS-CDS route does not. Therefore, EUS-CDS is expected to have a longer stent patency than ERCP. However, for first-line biliary drainage, it remains unclear whether EUS-CDS or ERCP is superior in terms of stent patency. To reduce the frequency of highly adverse events (AEs) such as bile peritonitis or stent migration following EUS-CDS, we developed an antimigration metal stent with a thin delivery system for tract dilatation. This study is designed to assess whether EUS-CDS with this novel stent is superior to ERCP with a traditional metal stent in terms of stent patency when the two techniques are used for first-line drainage of malignant distal biliary obstruction.

**Methods/design:**

This study is a multicenter single-blinded randomized controlled trial (RCT) involving 95 patients in four tertiary centers. Patients with malignant distal biliary obstruction that is unresectable or presents a very high surgical risk and who pass the inclusion and exclusion criteria will be randomized to EUS-CDS or ERCP in a 1:1 proportion. The primary endpoint is the stent patency rate 180 days after stent insertion. Secondary outcomes include the rates of technical success, clinical success, technical success in cases not requiring fistulous-tract dilation (only EUS-CDS group), procedure-related AEs, re-intervention success, patients receiving post-drainage chemotherapy, procedure time, and overall survival time.

**Discussion:**

If EUS-CDS is superior to ERCP in terms of stent patency and safety for the first-line drainage of malignant distal biliary obstruction, it is expected that the first-line drainage method will be changed from ERCP to EUS-CDS, and that interruption of chemotherapy due to stent dysfunction can be avoided.

**Trial registration:**

University Hospital Medical Information Network Clinical Trials Registry (UMIN-CTR), ID: UMIN000041343. Registered on August 6, 2020. https://upload.umin.ac.jp/cgi-open-bin/ctr_e/ctr_view.cgi?recptno=R000047201

Version number: 1.2, December 7, 2020.

## 1 Introducion

The criterion standard procedure for malignant biliary drainage is transpapillary drainage with endoscopic retrograde cholangiopancreatography (ERCP).^[1,2]^ However, in 20% to 40% of patients, the insertion of a metal stent through the tumor leads to subsequent stent dysfunction secondary to tumor tissue stent ingrowth and/or overgrowth,^[3,4]^ and EUS-guided biliary drainage (EUS-BD) has recently become the modality of choice when ERCP fails.^[5]^ One such EUS-BD method, which was first described in 2001,^[6]^ is EUS-guided choledochoduodenostomy (EUS-CDS), where an anastomosis is created between the duodenal bulb and the extrahepatic bile duct. Patients with distal biliary obstruction and normal gastrointestinal anatomy may be candidates for EUS-CDS, which is not subject to tumor tissue ingrowth or overgrowth of the stent, unlike transpapillary drainage with ERCP.

Some studies have reported comparisons of EUS-BD and ERCP in first-line biliary drainage. Paik et al^[7]^ reported an randomized controlled trial (RCT) that found EUS-BD to have a higher stent patency rate than ERCP (85% vs 49%, respectively), but 2 other RCTs reported that EUS-BD had comparable stent patency to ERCP^[8,9]^; the differences in stent patency between EUS-BD and ERCP therefore remains unclear.

**Figure 1:**
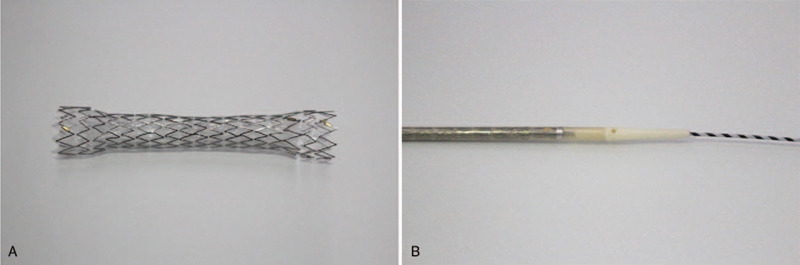
The stent and delivery system (Covered BileRush Advance, Piolax Medical Devices, Yokohama, Japan). (A) The expanded stent has a diameter of 8 mm and is 60 mm in length. It is made of laser-cut nitinol wire and is partially covered by a silicone membrane. A 5-mm section at the proximal end of the stent is uncovered and flared to a diameter of 10.5 mm to prevent distal migration. The distal end is also flared to a diameter of 10.5 mm to prevent inward migration. (B) This stent is delivered with a 7.0-Fr pull-back delivery catheter. The tip of the delivery system is shaped (2.6 Fr) to enable stent deployment without fistula dilation.

We previously developed a metal stent suitable for EUS-CDS (Covered BileRush Advance, Piolax Medical Devices, Yokohama, Japan; Figure 1)^[10]^ and reported a prospective multicenter study that showed EUS-CDS with this metal stent to have 95% technical and 100% clinical success rates, with procedure-related adverse events (AEs) reported in 5% of cases.^[11]^ Therefore, this study is designed to assess whether EUS-CDS with the novel stent offers superior stent patency to ERCP with a traditional metal stent when used as a first-line drainage method for malignant distal biliary obstruction.

## 2 Methods/design

### 2.1 Ethics approval and patient consent

This study is approved by the Wakayama Medical University Ethics Committee (No. 2958), and informed consent will be obtained from all patients. The trial is registered with the University Hospital Medical Information Network (trial registration no. UMIN000041343). This protocol was prepared in conformance with the Standard Protocol Items: Recommendations for Interventional Trials (SPIRIT) guidelines [see Additional file 1].

### 2.2 Study aims and design

This study is a multicenter prospective RCT across four tertiary centers in Japan, comparing first-line biliary drainage for malignant distal bile duct obstruction between EUS-CDS and ERCP (Fig. 1). We hypothesize that EUS-CDS with the novel stent is superior in terms of stent patency to ERCP with a traditional metal stent when the procedures are used as first-line drainage for malignant distal biliary obstruction. We expect that EUS-CD with the novel stent will allow avoidance of interruption to chemotherapy due to stent dysfunction, and will thereby ultimately improve survival time.

### 2.3 Patients

**Figure 2:**
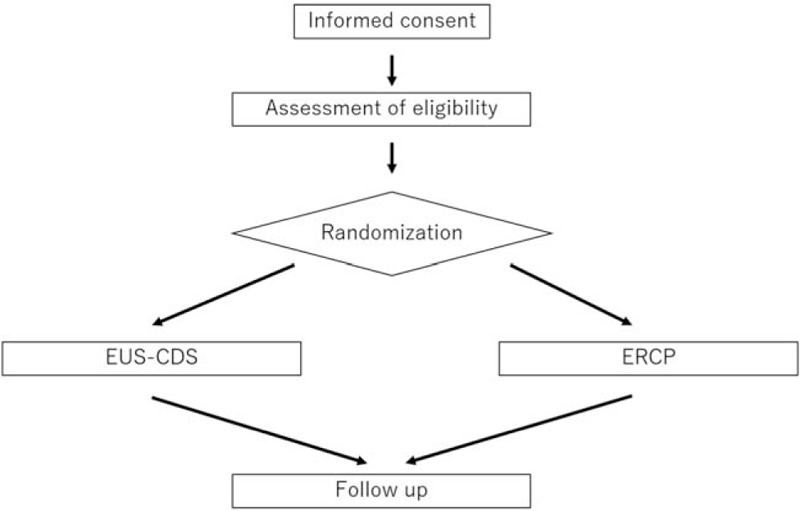
Flowchart of the study design.

At each center, the on-site study investigators will obtain informed consent from the candidates, and they will use an electronic data capture system to input necessary information, confirm that the candidates meet the eligibility criteria (ie, the candidates meet all the inclusion criteria and none of the exclusion criteria), and register the candidates with the registration secretariat. After confirming that a candidate meets the criteria, a registration number will be issued, and the registration will be considered complete. Patients who have completed the registration will be randomly assigned (1:1) to receive EUS-CDS or ERCP for relief of biliary obstruction. A flowchart of the study design is shown in Figure 2.

### 2.4 Inclusion criteria

The following inclusion criteria will be applied:

1. Age ≥ 20 years.
2. Radiological diagnosis (with or without pathological confirmation) of malignancy and presence of a malignant distal biliary obstruction and dilation of the upstream bile duct that is unresectable or presents a very high surgical risk.
3. Written consent obtained following adequate explanation of the study aims, design, and procedures.

### 2.5 Exclusion criteria

The following exclusion criteria will be applied:

1. Radiological evidence of gastric outlet obstruction on CT.
2. Hilar biliary obstruction.
3. Surgically altered anatomy.
4. ECOG performance status of 4.
5. Poorly controlled ascites.
6. History of endoscopic sphincterotomy (EST).
7. Biliary stenting.
8. Serious complication involving another organ.
9. Uncorrectable coagulopathy and/or thrombocytopenia.
10. Any other condition or situation determined by a study investigator to represent a reason for ineligibility.

### 2.6 Randomization and blinding

Subjects will be assigned to one of the treatment methods at a ratio of 1:1 by dynamic allocation according to a web-based registration program system. Based on the baseline factors for treatment allocation, including primary disease (pancreatic cancer or not) and institution is computed. The registration secretariat will strictly control the program to prevent leakage of the assignment information to other involved personnel. Blinding will not be used in this study.

### 2.7 EUS-CDS procedure

An echoendoscope is inserted orally and advanced to the duodenal bulb. Biliary accessibility is then confirmed via EUS from the duodenal bulb, with Doppler imaging used to rule out any intervening vessels. A 19-gauge fine aspiration needle is used to puncture the extrahepatic bile duct under endosonographic guidance. After the bile juice is aspirated, contrast medium is injected and a 0.025 inch guidewire is advanced into the common bile duct. Thereafter, an attempt is made to insert the novel stent (8 mm; Covered BileRush Advance, Piolax Medical Devices, Yokohama, Japan) via the fistula without using any dilation devices. If successful, the metal stent is expanded between the common bile duct and the duodenum under EUS guidance. If the delivery system cannot be advanced via the fistula, the fistulous tract is dilated using dilation devices. The stent diameter is 8 mm, and the stent length is determined at the discretion of the endoscopist.

### 2.8 ERCP procedure

ERCP-assisted transpapillary stenting is performed using a standard duodenoscope. Following biliary cannulation, contrast medium is injected to obtain a cholangiogram.

After confirmation of successful bile duct cannulation, biliary sphincterotomy is performed. Finally, a fully covered self-expandable metal stent (X-Suit NIR; Olympus Medical Systems, Tokyo, Japan) is placed across the papilla and the biliary stricture. The stent diameter is 10 mm, and the stent length is determined at the discretion of the endoscopist.

### 2.9 Primary endpoint

The primary endpoint is the stent patency rate at 180 days after stent insertion. The stent patency rate is defined as the percentage of patients who do not experience stent dysfunction from the date of stent insertion to 180 days after treatment.

Stent dysfunction is defined as follows:

1. If the lowest serum total bilirubin (T-Bil) value in the blood tests performed after treatment is <1.5 mg/dL: a serum T-Bil level of ≥3.0 mg/dL and a bile duct diameter greater than that on the pretreatment imaging.
2. If the lowest serum T-Bil value in the blood tests performed after treatment is more than 1.5 mg/dL: a serum T-Bil level >2.0 times the lowest value and a bile duct diameter greater than that on the pretreatment imaging.

Patients who are lost to follow-up or who die without stent dysfunction will be censored at the last observation date. Patients without technical success will be censored at the treatment date.

### 2.10 Secondary endpoints

The secondary endpoints are as follows:

1. Technical success rate: Technical success is defined as the stent being placed between the duodenum and common bile duct at 1 day after treatment in the EUS-CDS group, and the stent being placed across the papilla and the biliary stricture on CT at 1 day after treatment in the ERCP group.
2. Clinical success rate: Clinical success is defined as improvement in T-Bil to less than 1.3 mg/dL or improvement in T-Bil or alkaline phosphatase levels to <50% of the highest pretreatment values within 14 days after treatment.
3. Procedure-related AE rate: Early AEs (within 14 days after treatment) are defined according to the Cotton criteria^[12]^ and the ASGE lexicon for endoscopic AEs,^[13]^ and include postprocedural pancreatitis, cholecystitis, non-occlusion cholangitis, stent migration, stent deviation, liver abscess, bleeding, intestinal perforation, and abdominal pain. Late AEs (>14 days until 180 days after treatment) are defined according to the ASGE lexicon for endoscopic AEs, and include nonocclusion cholangitis, stent migration, and stent deviation.
4. Procedure time: Procedure time is defined as the time from the puncture of the bile duct to stent placement in the EUS-CDS group, and from the time of biliary cannulation to stent placement in the ERCP group.
5. Re-intervention success rate: Re-intervention success is defined as the placement of a new drainage tube at the fistula in the EUS-CDS group, and the placement of a new transpapillary drainage tube in the ERCP group.
6. Technical success rate in cases not requiring fistulous-tract dilation (only EUS-CDS group).
7. Overall survival time.
8. Rate of patients receiving post-drainage chemotherapy: The rate of post-drainage chemotherapy is defined as the percentage of patients who receive at least 2 courses of chemotherapy after drainage.

### 2.11 Data collection

Baseline assessment will be performed during the screening period. Basic information including sex, age, date of birth, date of obtaining informed consent, history of EST, presence of biliary stenting, and assessment of performance status will be recorded retrospectively. To ensure feasibility and safety, complete blood counts, hepatic and renal function tests, and biochemical tests will be performed during the screening period.

**Figure 3:**
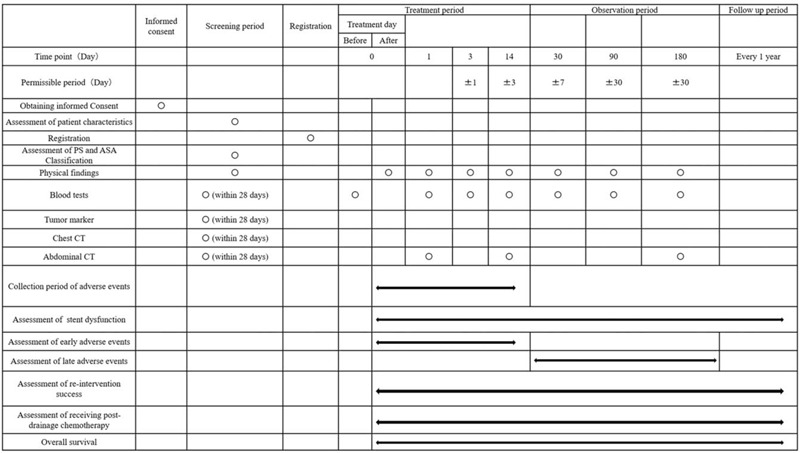
Standard protocol items (SPIRIT): schedule for data collection.

Computed tomography (CT) will be performed during the screening period to diagnose the tumor stage according to the TNM classification, to determine the site of biliary obstruction, and to determine the presence of gastric outlet obstruction. Patients will undergo the above-mentioned blood tests on post-procedure days 1, 3, 14, 30, 90, and 180 to evaluate the efficiency of the treatment, AEs, and stent dysfunction. CT will be performed postoperatively on days 1, 14, and 180 to evaluate the position of the stent, AEs, and stent dysfunction. The schedule for enrollment, interventions, and assessments is shown in Figure 3.

### 2.12 Follow-up

All patients will be followed until either death or 180 days after the treatment of the last registered patient. If the patient is unable to attend hospital, follow-up will be conducted over the phone. If follow-up information cannot be obtained, the patient will be considered lost to follow-up, and their data will be documented accordingly.

## 3 Statistical analysis

### 3.1 Sample size calculation

In this study, we assume that the stent patency rate at 180 days after stent insertion will be 85.1% in the EUS-CDS group and 48.9% in the ERCP group.^[9]^ In this case, assuming an exponential distribution for the survival time distribution, when the null hypothesis “stent patency rates at 180 days after stent insertion in the EUS-BD and ERCP groups are the same” is tested against the 2-sided alternative hypothesis “stent patency rates at 180 days after stent insertion in the EUS-BD and ERCP groups are different,” using a two-sample log-rank test at a significance level of α = 0.01 (2-sided), the number of events required to achieve a power of 1-βof 90% is 29. If the enrollment period is 12 months, the observation period after the last case enrollment is 6 months, and the death rate per month is 10.0% in both groups, then the minimum number of cases required is 90. Therefore, we have set a target number of 95 cases, assuming 5% are ineligible.

### 3.2 Statistical analysis

In the effectiveness analysis for the population, analysis of the full analysis set is the main analysis, and per protocol analysis is performed as a reference. The safety analysis for the target population will analyze all test examples. For the stent patency rate at 180 days after stent insertion, the *P* value for the difference between the two procedures will be calculated using a 2-sample log-rank test. The Kaplan–Meier method will be used to estimate survival curves for each group. Greenwood's formula will be used to estimate the confidence interval for the stent patency rate at 180 days after stent insertion, and Brookmeyer and Crowley's method will be used to estimate the confidence interval for the median rate. For the rates of technical success, clinical success, technical success in cases not requiring fistulous-tract dilation, procedure-related AEs, re-intervention success, and patients receiving post-drainage chemotherapy, the percentages, and 99% confidence intervals will be calculated for each group. For the procedure time, the mean and 99% confidence interval will be calculated for each group. For the overall survival time, the *P* value for the difference between the 2 groups will be calculated with a 2-sample log-rank test. A *P* value of ≤.01 will be considered statistically significant. All analyses will be performed using JMP Pro version 14 (SAS Institute, Inc., Cary, NC).

## 4 Adverse event reporting

### 4.1 Definition of an AE

An AE is defined as any unfavorable or unintended illness or disability and its manifestations occurring in a subject up to 14 days after the end of the protocol treatment, regardless of whether it is causally related to the protocol treatment. A clinically significant worsening of symptoms that existed before the study is also considered an AE. However, worsening of the primary disease is not included as an AE. Physiological changes that are not considered clinically important in terms of frequency or severity will not be considered as AEs. If an AE is observed in a subject, then the principal investigator (or sub-investigator) will immediately ensure the safety of the subject, take appropriate measures, and describe the event in the case report.

In principle, CTCAE v5.0 should be used for the evaluation of adverse events/adverse reactions. However, for the following events, diagnosis, and severity assessment should be performed according to the respective criteria.

1. Postprocedural pancreatitis: Cotton criteria.^[12]^
2. Cholecystitis, nonocclusion cholangitis, stent migration, stent deviation, liver abscess, bleeding, intestinal perforation, and abdominal pain: ASGE guidelines.^[13]^

### 4.2 Definition of a serious adverse event

A serious adverse event is an AE occurring during the procedure or any time after the procedure that fulfills ≥ of the following criteria:

1. Results in death
2. Is immediately life-threatening
3. Requires in-patient hospitalization or prolongation of existing hospitalization
4. Results in persistent or significant disability or incapacity
5. A congenital abnormality or birth defect

The principal investigator of the institution where the serious AE occurs should take appropriate measures, regardless of whether or not there is a causal relationship with the research, and should immediately report the details of the event to the head of the research institution and the principal investigator, in accordance with the regulations of the respective medical institution.

### 4.3 Monitoring

Visit monitoring will be performed once a year by an independent data monitoring committee. The monitoring committee will collect information on the status of accumulation, inclusion/exclusion criteria, serious AEs, and any other relevant information, and strive to provide feedback to participating institutions for early resolution if there are any problems. The monitoring committee will also report any serious AEs to the committee for efficacy and safety assessment.

## 5 Discussion

This multicenter single-blinded RCT study is designed to assess whether EUS-CDS with a novel stent shows superior stent patency to ERCP with a traditional metal stent when used for the first-line drainage of malignant distal biliary obstruction. This clinical trial will also compare the rates of technical success, clinical success, procedure-related AEs, re-intervention success, patients receiving post-drainage chemotherapy, and procedure and overall survival time.

Currently, the criterion standard procedure for malignant biliary drainage is transpapillary drainage with ERCP, whereas EUS-CDS is the modality of choice when ERCP fails in patients with distal bile duct obstruction. The major problem affecting transpapillary drainage with ERCP is stent dysfunction due to tumor tissue stent ingrowth and/or overgrowth.^[3,4]^ By contrast, stent tumor tissue ingrowth or overgrowth does not occur after EUS-CDS because the drainage route passes beside the tumor. Therefore, EUS-CDS is expected to have longer stent patency than ERCP. If stent patency rate is shown to be higher in EUS-CDS than in ERCP, it would be clinically significant because it would not only reduce the number of re-interventions, but could also affect oncologic outcomes. In patients with unresectable pancreatic cancer with a good performance status, there is a clear survival benefit attributable to FOLFIRINOX therapy^[14]^ and nab-paclitaxel plus gemcitabine therapy,^[15]^ which are delayed if re-interventions for biliary obstruction are needed.

Post-ERCP pancreatitis (PEP) is a major complication of ERCP.^[16–19]^ PEP is caused by injury to the pancreatic duct during insertion of a catheter, guidewire, or stent into the duodenal papilla. Treatment of PEP often takes a long time and delays the start or resumption of chemotherapy. By contrast, as EUS-CDS does not involve the duodenal papilla, it has little effect on the pancreatic duct and rarely leads to pancreatitis. However, a serious problem with EUS-CDS is the occurrence of AEs such as bile leakage and stent migration in 17.9% to 23.3% of cases.^[20,21]^ The main reasons for the high rate of AEs are the technical complexity of the procedure and the lack of dedicated equipment. Therefore, we developed a metal stent suitable for EUS-CDS (Covered BileRush Advance, Piolax Medical Devices, Yokohama, Japan).^[8]^ We also reported a prospective multicenter study that found that EUS-CDS with this metal stent was associated with 95% technical and 100% clinical success rates, with procedure-related AEs reported in only 5% of cases.^[8]^ In 31.6% (6/19) of procedures, the delivery system was successfully inserted into the bile duct without requiring a fistulous-tract dilatation device. This stent has two advantages over conventional stents. First, the delivery system, which comprises a thin shaft (7.5Fr.) and a tapered tip (2.6Fr.), facilitates successful insertion into the bile duct without the use of fistulous-tract dilation, thereby preventing bile leakage during the procedure. Second, the stent is made from laser-cut wire and has a flare structure at both ends, features that may help to prevent stent migration. EUS-CDS using this novel thin delivery system stent might cause fewer AEs than EUS-CDS with a standard stent, especially bile peritonitis and stent migration. Recently, the delivery system for this metal stent was further reduced in diameter (7.0Fr) to reduce the need for fistulous-tract dilation.^[7]^ This trial will use this thinner delivery system stent (7.0Fr), and it is therefore expected that it will be possible to insert the delivery system into the bile duct without the use of fistulous-tract dilation in a higher proportion of cases.

Recently, three RCT studies compared EUS-BD and ERCP in first-line biliary drainage.^[9–11]^ One study reported that EUS-BD had a higher rate of stent patency and a lower rate of procedure-related AEs than ERCP, while two studies reported that EUS-BD had comparable stent patency and AE rates to ERCP. Two of these three RCT studies were subject to the major limitations that they were single-center studies and included only a small number of patients.^[10,11]^ Although the other study included a larger number of patients, it was subject to the major limitation that it included techniques other than EUS-CDS (such as EUS-guided hepaticogastrostomy) in the EUS-BD group, and did not make an actual comparison between ERCP and EUS-CDS. We believe that our study will be superior to these three studies in that it is a multicenter study, it will include statistically sufficient sample sizes, and it will perform actual comparisons between ERCP and EUS-CDS, with the EUS-CDS being performed using the more suitable novel stent.

## 6 Conclusions

This study is designed to assess whether EUS-CDS with a novel stent is superior in terms of stent patency to ERCP when the procedures are used for first-line drainage of malignant distal biliary obstruction. If EUS-CDS is superior to ERCP for first-line drainage for malignant distal biliary obstruction in terms of stent patency and safety, it is expected that interruption to chemotherapy due to stent dysfunction and adverse effects will be avoided, and that EUS-CDS may replace ERCP as the first-line drainage method.

## Author contributions

MI, MK, MT, TO, AM and HS conceived the study, designed the study protocol, and drafted the manuscript. MI and MK wrote the manuscript. RA, YY, KH, TY, NN and AS are in charge of coordination and direct implementation. TY and TS helped to develop the study measures and analyses. All authors contributed to drafting this study protocol manuscript and have read and approved the final version.

**Conceptualization:** Masahiro Itonaga, Masayuki Kitano.

**Data curation:** Masahiro Itonaga, Reiko Ashida, Yasunobu Yamashita, Kenichi Hatamaru, Mamoru Takenaka, Tomohiro Yamazaki, Takeshi Ogura, Nobu Nishioka, Arata Sakai, Atsuhiro Masuda, Hideyuki Shiomi.

**Formal analysis:** Takanori Yoshikawa, Toshio Shimokawa.

**Funding acquisition:** Masahiro Itonaga.

**Investigation:** Reiko Ashida, Yasunobu Yamashita, Kenichi Hatamaru, Mamoru Takenaka, Tomohiro Yamazaki, Takeshi Ogura, Nobu Nishioka, Arata Sakai, Atsuhiro Masuda, Hideyuki Shiomi.

**Methodology:** Masayuki Kitano.

**Writing – original draft:** Masahiro Itonaga.

**Writing – review & editing:** Masayuki Kitano.
